# Comparison of the Level of Awareness about the Transmission of Echinococcosis and Toxocariasis between Pet Owners and Non-Pet Owners in Greece

**DOI:** 10.3390/ijerph17155292

**Published:** 2020-07-22

**Authors:** Christina Kantarakia, Maria E. Tsoumani, Antonis Galanos, Alexander G. Mathioudakis, Eleni Giannoulaki, Apostolos Beloukas, Chrysa Voyiatzaki

**Affiliations:** 1 Laboratory of Molecular Microbiology & Immunology, Department of Biomedical Sciences, University of West Attica, 12243 Athens, Greece; ml12113@uniwa.gr (C.K.); mtsoumani@uniwa.gr (M.E.T.); egiannoul@uniwa.gr (E.G.); abeloukas@uniwa.gr (A.B.); 2 Laboratory of Research of the Musculoskeletal System, School of Medicine, University of Athens, 12243 Athens, Greece; galanostat@yahoo.gr; 3 Division of Infection, Immunity and Respiratory Medicine, School of Biological Sciences, The University of Manchester, Manchester M23 9LT, UK; Alexander.Mathioudakis@Manchester.ac.uk; 4 Institute of Infection and Global Health, University of Liverpool, Liverpool L 69 7BE, UK

**Keywords:** awareness, cats, dogs, echinococcosis, parasites, pet owners, toxocariasis

## Abstract

Ζoonotic parasitic diseases that can occur through animal contact pose risks to pets, their owners and to their bond. This study aims to assess the level of knowledge about zoonoses, specifically echinococcosis and toxocariasis, among cat/dog owners and non-pet owners in Greece. Multiple-choice questionnaires were designed to obtain data regarding the knowledge of pet and non-pet owners on echinococcosis and toxocariasis, including signs and symptoms of these zoonoses, ways of transmission and precautions that need to be taken into account in order to avoid it. A total of 185 questionnaires were retrieved and data was expressed as absolute (Ν) and relative frequencies (%). Associations between pet ownership, residence and outcome variables were evaluated using the Fisher exact test and Chi-squared test, respectively. Multifactorial linear regression analysis was used to investigate the cross-sectional association between demographic characteristics and the awareness of helminthic zoonoses. All tests were two-sided and statistical significance was set at *p* < 0.05. Our study revealed a disturbing lack of awareness of echinococcosis and toxocariasis (mean zoonotic knowledge score 8.11 ± 3.18) independently of pet ownership. Surprisingly, in some cases the ignorance of pet owners exceeded that of non-pet owners. Given the progressive impact of toxocariasis in public health and the high prevalence of echinococcosis in the Mediterranean region, measures should be taken to inform people about zoonoses and eliminate their putative transmission.

## 1. Introduction

Nowadays, many households own at least one pet, with dogs and cats being the most common ones, followed by fish, birds, rabbits and hamsters [1]. Humans and pets develop a strong emotional relationship, known as the “human-animal bond”, and many studies support the benefits it offers in terms of socialization, physical and mental health [2,3]. Additionally, it is widely accepted that pets also contribute to improving recovery rates for a large number of diseases such as cardiovascular or respiratory problems, anxiety and depression [4]. However, this close relationship between animals and humans may result in an increased risk of exposure to infectious diseases since pets are a potential source for more than 60 zoonotic agents [5]. In fact, dogs and cats play a major role in spreading zoonoses such as echinococcosis and toxocariasis, which are transmitted directly from pets to the human environment, without involving vectors or other intermediate hosts [1,2,3]. 

Human toxocariasis, a neglected parasitic infection, can develop serious syndromes, known as visceral larva migrans, ocular larva migrans, neurotoxocariasis, and covert or common toxocariasis. Following accidental ingestion of infective eggs containing third stage larvae of roundworms *Toxocara cati* and *Toxocara canis*, or larvae in under-cooked infected organ or muscle tissues (rare), Toxocara larvae hatches, penetrates the intestinal mucosa and migrates via the blood circulation to various tissues (e.g., liver, heart, brain, lungs, skeletal muscle and eyes) causing local inflammation and granuloma [6,7]. Visceral larva migrans (VLM) is the most common syndrome in infected people, particularly children. Although most infections are light, with clinical signs such as fever, pulmonary congestion and eosinophilia, in heavy infections some people develop lymphadenopathy, granulomatous hepatitis, nephritis, and arthritis, elevation of serum immunoglobulin E (IgE)concentration, presence of allergen-specific IgE, asthma and promotion of pulmonary fibrosis [8]. Ocular larva migrans (OLM) commonly reported in children 3–16 years of age, can be caused by even a single *Toxocara* spp.

Larva become entrapped in the eye and characterized by significant visual disability, photophobia, retinitis, granulomata, and blindness [9]. Neurotoxocariasis, a rare syndrome in middle-aged people, relates to the migration of *T. canis* larvae in the Central Nervous System with clinical signs of meningitis, encephalitis and myelitis [10]. Covert toxocariasis in children or common toxocariasis in adult people is characterized by nebulous symptoms such as fever, headache, vomiting, nausea, abdominal pain, lymphadenitis, hepatomegaly and pulmonary dysfunction [7]. Human echinococcosis is a parasitic zoonotic disease, caused by the larval form of taeniid cestodes *Echinococcus granulosus sensulato* (cystic echinococcosis), *E. multilocularis* (alveolar echinococcosis), *E. vogeli* and *E. oligarthus* (polycystic echinococcosis). The two most important forms, which are of medical and public health relevance in humans, are cystic echinococcosis and alveolar echinococcosis. The clinical potential of two other *Echinococcus* species (*E. shiquicus* and *E. felidis*) is unknown [11]. Echinococcosis is contracted by accidental ingestion of fully developed infectious eggs (containing an oncosphere larva) from the feces of dogs or other carnivores, with humans serving as intermediate hosts instead of sheep, cattle, mice or other herbivores as well as pigs. It must be noted that the eggs excreted by defecation can be dispersed from the deposition site either by being washed away or carried by flies and other vectors. Additionally, *Echinococcus* eggs may be widely dispersed by adhering to tyres, shoes or animal paws, resulting in contamination of the environment, including human dwellings [12]. Hydatids or larval cysts are formed in many tissues, producing lesions and symptoms of which the incubation period and clinical picture depends on the location, number and state of the cyst, the tissues affected etc. The liver, the most commonly invaded organ, may take twenty years to present symptoms, such as jaundice. The lungs, mediastinum, peritoneum, kidneys, spleen, vertebral column and brain, may also be affected, producing symptoms ranging from respiratory distress and kidney dysfunction to seizures. Hydatid cyst rupture can produce anaphylactic shock. Eosinophilia accompanies the infection [13]. It is worth noting that the World Health Organization (WHO) has listed echinococcosis as one of the 17 neglected diseases targeted for control or elimination by 2050 [14].

Awareness of both echinococcosis and toxocariasis, and their transmission to humans, attaches great importance to the design of elimination plans and prevention strategies. Prevention requires an ‘One Health’ approach, persisting training of veterinarians and continuing education of pet owners [12]. The support of education programs that promote adopting healthy practices (for instance, not feeding raw offal to dogs in order to prevent cystic echinococcosis) is important for the success of zoonotic disease control programs [15].

Evidence from several studies suggest that awareness of the perception of the zoonotic potential of some parasites by pet owners is limited. A study published in 2010 reported that only 56% of dog owners in Texas, USA were cognizant of the zoonotic potential of intestinal helminthes [16]. Similar results were derived in the most recently conducted study in Portugal in 2016 [17]. In a study conducted in 2012 in Ontario, Canada, where questionnaires were also distributed, only 27% of the responders recalled being informed by a veterinarian about zoonoses [1]. Finally, a study carried out in 2008 in Nigeria, using a survey, revealed an inadequate knowledge of parasitic diseases in dogs, in spite of the high prevalence of ectoparasites (60%) and intestinal helminths (68%) in the region. Public information regarding zoonoses was poor and the diseases were not considered of high concern as a health issue [18].

There are several, mainly epidemiological, studies concerning cases of these particular helminthic infestations in Greece. A seroepidemiological investigation of *Toxocara canis* in a female Greek pregnant population in the area of Athens in 2016 concluded that the prevalence of *Toxocara canis* infection in a population of Greek pregnant women was found to be at a rate of 17% and the soil contamination rate was 17% [19]. In another seroepidemiologic survey, involving children, a remarkably high percentage (13%) reacted positively to this method. Sixteen (3%) out of 511 sera showed immunoglobulin G (IgG) antibodies, 43 (8%) showed immunoglobulin M (IgM), while 5 (1%) showed both IgG and IgM antibodies against *T. canis* excretory–secretory (ES) antigen. Females were significantly more infected than males. Seropositivity rate was highest in children over the age of 10 and was not found to differ significantly with age (*p* ≥ 0.05). Males and females were found to differ significantly only in the sero-prevalence of IgM antibodies in the first age group (less than 5 years old) [20]. In addition, in a study published in 2018, concerning soil contamination in the Attica region, *T. canis* eggs were isolated from 31 (94%) of the examined public areas. Of the total samples, *T. canis* ova were recovered from 258 samples, suggesting an overall *T. canis* ova contamination of 17%. The areas of higher socioeconomic status presented lower percentages of soil contamination at a statistically significant level, compared to the areas of lower socioeconomic status. *T. canis* IgG seropositivity was detected in 40 (16%) serum samples. Similar rates were established among *T. canis* seropositivity and soil contamination within the same geographical areas. The proportion of seropositive samples in the group of children was significantly higher compared to the proportion of adults (48% versus 8%, *p* < 0.001) [21]. In Crete, Greece, totals of 879 dog and 264 cat fecal samples were examined in 2017. In dogs, the overall prevalence was 0.8% (CI: 0.2–1.4) for taeniid eggs. In cats, the prevalence was 8.3% (CI: 5.0–11.7) for *Toxocara* spp.; 0.8% (CI: 0–1.8) for taeniid eggs [22]. A review gathering incidents of cystic echinococcosis in Northern Greece reports that in the 1970s, 123 cases of cystic echinococcosis were dealt with; in the 1980s, the number of cases decreased to 54; in the period between 1990 and 2003, cases decreased to 8; and in the period 2004–2009, there were 2 recorded cases. This listing referred to children ranging from 2 to 14 years, with an average age of 9.2 years. The youngest patient was 23 months of age [23].

Nevertheless, to the best of our knowledge, a survey study assessing awareness and knowledge of these parasitic zoonoses has never been conducted in Greece, despite the higher risk of echinococcosis in the Mediterranean region [24,25] and the high rates of environmental contamination with *Toxocara* eggs in Europe [26]. The aim of this study was to evaluate the awareness and knowledge of cat and/or dog owners in Greece regarding the zoonotic potential of helminthic parasites, especially *Echinococcus* spp. and *Toxocara* spp., which dogs and cats can harbor, in comparison to non-pet owners.

## 2. Materials and Methods 

### 2.1. Survey Protocol and Design

From September to October 2018, we distributed anonymous self-administered, primarily multiple-choice paper questionnaires at the University of West Attica, in Athens, the Technological Educational Institute (TEI) of Thessaly in Larissa, and via the internet, through an anonymous questionnaire platform. The questionnaire, which consisted of 3 pages, was developed with the cooperation and guidance of biomedical scientists, veterinarians and statisticians (see Supplementary Material). The questionnaire collated information such as demographics (i.e., gender, age, residency and education level), participants’ knowledge of echinococcosis and toxocariasis, their signs and symptoms, consequences and therapy, participants’ sources of information, pet and generally animal contact-related attitudes, types of pet owned (if any), reported cases of echinococcosis or toxocariasis, knowledge about ways of transmission and precautions that need to be taken into account in order to avoid them. Pet owners were asked to enlist their pets’ residence as ‘only indoors’, ‘only outdoors or ‘combined’. The use of these categories in the questionnaire was based on previous studies [1,17,27]. The study was approved by the Research Ethics Board of the University of West Attica (ref 3/11.02.2016). 

### 2.2. Statistical Data Analysis

Demographic and other data are summarized using absolute and relative frequencies. Simple comparisons of the relevant distributions across different levels of other categorical variables are based on chi-square tests. Associations between pet ownership, residence and outcome variables were evaluated using the Fisher exact test and Chi-squared test, respectively. Unifactorial analysis was performed using the One way Analysis of Variance model with the Bonferroni test for pairwise comparisons. Multifactorial linear regression analysis was used to investigate the cross-sectional association between demographic characteristics and the awareness of helminthic zoonoses. All variables were included in the model, using the Enter method, to determine the influence of independent predictors on awareness of helminthic zoonoses. All assumptions of linear regression analysis (homoscedasticity, linearity, normality and independence of error terms, as well as multicollinearity of independent variables) were examined. All tests were two-sided and the statistical significance was set at *p* < 0.05. All analyses were carried out using the statistical package SPSS version 17.0 (Statistical Package for the Social Sciences, SPSS Inc, Chicago, IL, USA).

## 3. Results

Out of a total of 200 respondents, 15 were excluded from the study because they owned rodents or rabbits as pets and not cats or dogs and this may have affected their answers. Consequently, data analysis was restricted to only dog and/or cat owners and non-pet owners, in a total of 185 (93%) respondents.

The rate of dog or cat owners and non-pet owners of those responded the questionnaire was almost equally shared, as 55% of them owned at least one pet (dog or cat) and 45% were non-pet owners. The majority of the respondents were women (77%). Most of the participants were young (86% were between 18 and 39 years old), highly educated (83%—university as education level) and lived in a city (74%). The majority of pet owners had dogs as pets (67%) whereas 17 of the respondents owned both cat(s) and dog(s). Of the 101 pet owners, 50% kept their pet(s) indoors, 21% kept them outdoors while 35% of them kept them both indoors and outdoors (Supplementary Table S1).

Regarding the social perception of zoonoses, the majority reported that they were somewhat concerned about contracting a disease, followed by those who were not at all concerned (54% and 23%, respectively). The percentages of the answers concerning the sources of information about zoonoses were almost evenly shared, with veterinarians and the internet being the most frequent answers (22% and 21%, respectively). Forty-seven of the 185 participants (25%) were satisfied with their knowledge about zoonoses, and a high proportion (45%) replied that they were satisfied with their awareness of the precautions needed to avoid the diseases’ transmission. Only five respondents reported that there was a case of echinococcosis or toxocariasis in their close circle (Table 1).

Furthermore, we assessed the knowledge of the respondents concerning echinococcosis and toxocariasis. Total scores can range from 0 to 15, with higher scores indicating a higher degree of knowledge. Overall, participants had a middle level of consciousness of these two helminthic zoonoses (mean score ± SD; 8.11 ± 3.18). The zoonotic disease knowledge score did not differ between pet and non-pet owners (Table 2). It is worth noting that unifactorial analysis revealed that people who graduated from University or College presented higher levels of awareness of helminthic zoonoses compared with those who graduated from High school. All other variables (including ownership) did not affect the dependent variable (Table 2).

In Table 3, we report the answers of the participants, most of which were used for calculating the mean zoonotic disease knowledge score (please see the appendix provided as Supplementary Material). Most of the respondents answered correctly that consumption of raw/uncooked food from pets enhances the probability of infection (75%). Eighty-three of the participants (44.9%) knew that immunosuppressed people are more vulnerable to zoonoses, while 78 of them (42%) reported that they did not know the right answer. More than a half of the participants declared ignorance when asked whether the inhabitants of the Mediterranean region have an increased risk of echinococcosis and the same answer’s rate was also high when they were asked about the appropriate treatment for echinococcosis and toxocariasis (37% and 50%, respectively). In addition, 65.4% of the respondents think that the risk of transmission for these zoonoses is raised after contact with soil in areas accessible to dogs/cats. Approximately one-third out of a total of 101 pet owners claimed that they deworm their pet(s) every 6 months. Regarding the questions enquiring specifically about the transmission, high risk age group, and affected organs from the zoonoses, there was a great percentage that reported unfamiliarity for both zoonoses, with that choice gathering the highest rate in many of them (Table 3).

Subsequently, we assessed the level of awareness regarding echinococcosis and/or toxocariasis transmission and prevention (Table 4). The two most common ways of transmission of the zoonoses, i.e., consumption of contaminated food/water and through the fecal–oral route, were the ones that marked the lowest rates of correct answers (Table 4). Importantly, the level of awareness regarding transmission was similar between pet and non-pet owners (Table 4). On the contrary, high rates of correct answers about precautions were noted, with exception of the answer for the vaccination of pets, where participants provided the wrong answer, meaning that vaccination of pets is a way that can be used for prevention (Table 4).

The percentages of correct answers regarding the zoonotic disease knowledge and animal contact-related attitudes of respondents are shown in Table 5. Despite the fact that there were no statistically significant differences among pet and non-pet owners, it is important to highlight the very low rates of correct answers in the majority of these questions. Finally, no statistically significant results were found in the responses of pet owners in relation to the pet’s place of residence (inside, outside or mixed) (Supplementary Table S2).

Finally, we employed a multiple regression model with the Enter method in order to examine the independent contribution of demographic variables to the score of awareness of helminthic zoonoses. Regression analysis accounted for 10% of the variance of the dependent variable [R^2^ = 0.101, F(5179) = 3.6, *p* = 0.004]. According to our results, higher levels of Education (Beta coefficient ± SE: 2.38 ± 0.65; *p* < 0.005; R^2^ = 0.09) were statistically significantly associated with higher awareness of helminthic zoonoses, while Age (Beta coefficient ± SE: 0.06 ± 0.56; *p* = 0.695; R^2^ = 0.001), Gender (Beta coefficient ± SE: −0.28 ± 0.72; *p* = 0.695; R^2^ < 0.001), Location of Residence (Beta coefficient ± SE: −0.41 ± 0.53; *p* = 0.443; R^2^ = 0.004) and Pet-ownership (Beta coefficient ± SE: 0.11 ± 0.47; *p* = 0.822; R^2^ < 0.001) did not have a statistically significant influence on the dependent variable (Table 6).

## 4. Discussion

Echinococcosis and toxocariasis constitute some of the most important and common zoonotic infections threatening human populations in Europe [12]. We aim to assess the knowledge of cat and/or dog owners about echinococcosis and toxocariasis, in comparison to non-pet owners, in addition to their beliefs and attitudes with respect to pet ownership, in Greece. Although this subject is of major significance for public and personal health, a similar study has never been carried out in Greece. 

We found a poor knowledge about both parasites, regardless of whether respondents were pet owners, or not. Similarly, in a study conducted in 2016 in Portugal, it was noted that 56.5% of the pet owners were familiar with zoonoses as a term, but only 35.2% understood what it means. However, the higher the educational level, the better the understanding about zoonoses was [19]. This is consistent with our results showing that education is the only variable that significantly affects the zoonotic parasitic knowledge score.

Regarding *Toxocara* spp., in our study, the rates of correct answers in most of the questions were very low. In a recent study, a high percentage of pediatricians stated that they were not confident that their knowledge of toxocariasis was updated. In addition, the majority of respondents could not discriminate between toxoplasmosis and toxocariasis and also, they were not able to correctly identify prevention strategies to decrease risks of acquiring *Toxocara* infection. In an effort to combat this gap, the Centers for Disease Control and Prevention (CDC) have developed downloadable resources for the public and for physicians, available at https://www.cdc.gov/parasites/toxocariasis/printresources.html [28].

In our study, veterinarians (21.6%) and the internet (20.7%) were the most frequently reported source of information about zoonoses, which is consistent with a similar previously conducted study [1]. In a similar survey conducted in 2012 in Ontario, Canada, where questionnaires were also distributed, only 27% of the responders recalled having been informed by a veterinarian about zoonoses and over 30% of them were not concerned about catching a disease from their pets [1].

Veterinarians could be the most important educators regarding the transfer of knowledge of zoonoses to stakeholders. However, it seems that they hesitate to provide this information to their clients to avoid alarming them and leading them to give up their pets. In fact, a national survey of intestinal parasites in dogs and cats in Australia revealed that most veterinarians would not discuss with their clients the zoonotic risk of gastrointestinal parasites their pets may bear, resulting in the owners’ nescience. Nevertheless, they suggested regular anthelminthic treatment throughout a pet’s life. Moreover, pet owners were unaware of the existence of zoonoses [29].

We found a considerably low level of satisfaction about the knowledge of zoonoses (25.4%). It seems that respondents are not properly using the sources of knowledge, highlighting the imperative need for awareness campaigns among pet owners in Greece.

It should be mentioned that during the distribution of the questionnaires, supplementary questions (e.g., about their knowledge on which diseases are capable of being transmitted to humans through contact with cats and dogs) were made during the discussions with those interested in participating in the study. It is worth noting that there were respondents thinking that it is possible to be infected with HIV (Human Immunodeficiency Virus) (three participants) and hepatitis B virus (HBV) (seven participants) through animal contact.

Since the adequate knowledge of zoonotic diseases, and particularly echinococcosis that is endemic in Mediterranean region [30], is key to preventing them from spreading, the limited awareness is highly concerning. It is also crucial to mention that a heavily poor acknowledgement of the increased localization for these particular zoonoses in the Mediterranean Basin was observed, with only 27 of the 185 participants being cognizant of the elevated risk. In addition, the most common ways of transmission of echinococcosis and toxocariasis (fecal–oral route and consumption of contaminated food/water) marked the lowest rates of correct answers. It seems that there is a gap between owners’ and non-owners’ answers in whether the avoidance of infected people is useful in the prevention of helminthic transmission. Last but not least, almost 70% of the respondents erroneously believe that vaccination contributes to the eradication of these parasites.

The rate of correct answers was significantly decreased in questions regarding organs affected, the pet involved in the transmission, most vulnerable age group, treatment and deworming frequency. Veterinarians could play a critical role in the attempt to increase pet owners’ understanding of the importance of frequent deworming. In addition, the need to properly dispose of their pet’s feces will also help to highlight toxocariasis [31]. The most recent European Scientific Counsel Companion Animal Parasites (ESCCAP) guidelines provide research-based independent advice regarding risk factors and recommended deworming frequency [32]. In the literature, it has been reported that compliance to veterinary and guideline advice is poor, resulting in wrong decisions on behalf of pet-owners regarding routine deworming [33,34]. Indeed, very recently, it has been shown that pet-owners from five countries in Europe (France, Germany, Spain, Sweden and the UK) do not deworm their dogs and cats as frequently as is recommended by ESCCAP or required in order to reduce zoonotic risk and improve pet health [35].

It is surprising that the results obtained from the comparison of dog or/and cat owners and non-pet owners indicate no statistically significant difference among these two groups. In contradiction to what was expected, there were many questions whose results presented an exceeding ignorance of pet owners, since their rates of incorrect answers were higher compared to those of non-pet owners (e.g., the pet contributing to the spread of the disease, ways of transmission and precautions, etc.). In deworming frequency, although it is not statistically different between the pet’s residence, the percentage is quite low apropos the right answers.

Unlike the livestock parasites, the anthelmintic drug resistance in companion animals’ parasites has been detected to be of slow emergence, probably due to individual or small numbers in animal keeping, correct dosage to dogs and cats on a body weight basis and appropriate frequency of deworming. However, there are the exceptions of anthelmintic resistance of larvae *Dirofilaria immitis* [36,37], the proved anthelmintic resistance that had evolved in the canine hookworm *Ancylostoma caninum* [38,39], as well as praziquantel resistance in the zoonotic cestode *Dipylidium caninum* [40]. As yet, there are no reports confirming antihelmintic resistance in *Toxocara* species, or in other dog or cat internal parasite species. However, the increased frequency or underdosing of anthelmintic treatment could favor selection pressure for resistance, like in the case of the shelters and breeding kennels, where there may be concomitant administration of the same antiparasitic drug to all the dogs or cats [41].

Last but not least, taking into account the sad truth that Greece is a country with a major issue of stray animals, it is a matter of urgency for medical care programs to be carried out regularly (vaccinations, deworming, etc.). In Greece, ESCCAP guidelines are not widely known and thus not followed, highlighting that the veterinarians should take the responsibility to educate pet owners about the transmission routes of zoonotic parasites, as well as prevention and parasite control practices [12]. Conclusively, it is of paramount importance for the country to initiate educational campaigns about zoonoses and precautions, transmission and treatment, and for veterinarians to inform pet owners about the importance of appropriate antihelminthic treatment and monitor the frequency in order to avoid belated administration.

Our survey has the limitation of a restricted sample size since it was retrieved mainly from two geographic areas (Attica and Larissa). From the data analysis, it was observed that the participants were mainly young adults and, despite the easy access to many sources of information, we observed a lack of knowledge and awareness. Further investigation is required in order to see if these results will be confirmed in different populations. 

## 5. Conclusions

In conclusion, this particular study aimed to demonstrate the knowledge and attitudes with respect to pet ownership and zoonotic diseases in Greece. As occurred from the study, awareness regarding the risk of contamination with the parasites *Echinococcus* spp. and *Toxocara* spp. was very limited even among pet owners. In fact, there was no statistically significant difference between pet owners and non-pet owners’ knowledge concerning the parasites. It is an undeniable fact that the acknowledgement of ways of transmission and risk factors can prevent the perpetuation of such zoonoses. Precautions, such as more active involvement of veterinarians in informing pet owners of zoonoses, should be applied or enhanced. In addition, introduction of the matter in the educational system is a necessity, since misinformation or ignorance is dangerous, both for animals and humans. Moreover, campaigns should be held, for instance television spots, leaflets in vet clinics, pet shops and physicians’ offices, informing individuals about zoonoses with specific information about which diseases are considered zoonoses, their transmission, risk factors and necessary preventive measures in order to eliminate the spread without causing panic and mistreatment towards animals.

## Figures and Tables

**Table 1:** Social perception of zoonoses.

Question	Answers					
Q1. How concerned are you that the pet(s) in your family/social environment could transfer a disease to you or your family/friends?	Very concerned	Concerned		Somewhat concerned	Not at all concerned	
	11	32		100	42	
	5.9%	17.3%		54.1%	22.7%	
Q2. Sources of information about the diseases that may occur through animal contact (multiple answers allowed)	Family- friends	Veterinarian	Medical Staff	Internet	Books	Other
	85	124	71	119	93	83
	45.9%	67.0%	38.4%	64.3%	50.3%	44.9%
Q3. Are you satisfied with the comprehension and the knowledge about the diseases that can occur through animal contact?	No	Yes		I don’t know		
	81	47		57		
	43.8%	25.4%		30.8%		
Q4. Are you satisfied with the knowledge about the precautions that need to be taken in order to minimize the risk of disease transmission that can occur through animal contact?	No	Yes		I don’t know		
	37	84		64		
	20.0%	45.4%		34.6%		
Q5. Has anyone in your family/inner circle ever been infected with echinococcosis or toxocariasis?	No			Yes		
	180			5		
	97.3%			2.7%		

All variables are presented as absolute (Ν) and relative frequencies (%).

**Table 2:** Unifactorial analysis of knowledge of the zoonoses echinococcosis and toxocariasis.

		*N*	Mean ± SD	*p*-Value
Gender	Male	43	7.78 ± 3.20	0.450
	Female	142	8.20 ± 3.18	
Age	18–29	128	8.38 ± 3.20	0.123
	30–39	31	7.93 ± 3.35	
	40+	26	7.00 ± 2.74	
Education	High School	32	6.06 ± 3.09	<0.005
	University/College	153	8.54 ± 3.05	
Location of Residence	City	137	8.26 ± 3.19	0.283
	Other	48	7.68 ± 3.16	
Pet-owner	No	84	8.25 ± 3.23	0.589
	Yes	101	8.00 ± 3.16	
Pet	Dog	68	8.00 ± 3.30	0.720
	Cat	16	8.47 ± 2.90	
	Both	17	7.72 ± 2.92	

“Other” in the Location of residence includes: Suburban area (*n* = 21)/Village (*n* = 11)/Island (*n* = 16).

**Table 3:** Investigation of the population’s knowledge about the zoonoses echinococcosis and toxocariasis.

Question	Answers					
Q1. Consumption of raw or insufficiently cooked food from pets increases the risk of echinococcosis?	No		Yes		I don’t know	
	7		139		39	
	3.8%		75.1%		21.1%	
Q2. Are the immunosuppressed people (due to disease, syndrome, radiation or chemotherapy etc.) more susceptible to echinococcosis and/or toxocariasis?	No		Yes		I don’t know	
	24		83		78	
	13.0%		44.9%		42.1%	
Q3.The inhabitants of the Mediterranean region have increased risk of developing echinococcosis?	No		Yes		I don’t know	
	54		27		104	
	29.2%		14.6%		56.2%	
Q4. Children’s contact with soil/sand in areas accessible to dogs/cats increases the risk of echinococcosis and/or toxocariasis?	No		Yes		I don’t know	
	23		121		41	
	12.4%		65.4%		22.2%	
Q5. How frequently should dogs and cats be dewormed?	Monthly		Every 3 months		Every 6 months	Every 12 months
	19		28		32	22
	18.8%		27.7%		31.7%	21.8%
Q6. Contact with which animal is more likely to lead to the development of cystic echinococcosis in humans?	Both cats and dogs		Cat		Dog	I don’t know
	56		23		52	54
	30.3%		12.4%		28.1%	29.2%
Q7. Which age group has the greatest risk of developing echinococcosis?	Children		Adults	Elderly	All ages	I don’t know
	40		0	9	89	47
	21.6%		0 %	4.9%	48.1%	25.4%
Q8. Which organs/ tissues of humans may be damaged in cases of echinococcosis?	Lungs		Liver	Eyes	Brain	I don’t know
	53		41	13	28	50
	29.0%		22.0%	7.0%	15.0%	27.0%
Q9.Which is the appropriate treatment of echinococcosis?	Medication		Surgical Removal		Combination	I don’t know
	53		11		52	69
	29.0%		6.0%		28.0%	37.0%
Q10. The contact with which animal is more likely to lead to development of toxocariasis in humans?	Both cats and dogs		Cat		Dog	I don’t know
	45		58		11	71
	24.3%		31.4%		5.9%	38.4%
Q11. Which age group has the greatest risk of developing toxocariasis?	Children	Adults	Elderly		All ages	I don’t know
	38	5	6		69	67
	20.5%	2.7%	3.2%		37.3%	36.2%
Q12.Which organs/ tissues of humans may be damaged in cases of toxocariasis?	Lungs	Liver	Eyes		Brain	I don’t know
	26	41	32		33	53
	14.0%	22.0%	17.0%		18.0%	29.0%
Q13.Which is the appropriate treatment of toxocariasis?	Medication		Surgical Removal		Combination	I don’t know
	61		4		28	92
	33.0%		2.0%		15.0%	50.0%

All variables are presented as absolute (Ν) and relative frequencies (%).

**Table 4:** Participants’ awareness regarding echinococcosis and/or toxocariasis transmission and prevention in relation to pet ownership.

Question	Possible Options	Correct Answer	Total *N* (%)	Pet Owners (*n* = 101)	Non Pet Owners (*n* = 84)	*p*-Value
Q1. Possible way(s) of echinococcosis and/or toxocariasis transmission	a. Through fecal–oral route	Yes	136 (73.5%)	73 (72.2%)	63 (75.0%)	0.314
	b. Consumption of contaminated food/water	Yes	127 (68.6%)	67 (66.3%)	60 (71.4%)	0.201
	c. Sexual intercourse	No	176 (95.1%)	98 (97.0%)	78 (92.8%)	0.733
	d. By blood	No	165 (89.2%)	91 (90.1%)	74 (88.0%)	0.479
	e. Contact with infected person	No	165 (89.2%)	94 (93.0%)	71 (84.5%)	0.636
	f. I don’t know	*-*	158 (85.4%)	85 (84.1%)	73 (86.9%)	0.802
Q2. Precautions for preventing the development/transmission of echinococcosis and/or toxocariasis	a. Vaccination of individual	No	138 (74.6%)	76 (75.2%)	62 (73.8%)	0.614
	b. Vaccination of pet	No	58 (31.4%)	33 (32.6%)	25 (29.7%)	1
	c. Deworming of pet	Yes	125 (67.61%)	74 (73.2%)	51 (60.7%)	0.269
	d. Observance of personal hygiene rules (frequent handwashing etc.)	Yes	152 (82.2%)	85 (84.1%)	67 (79.7%)	1
	e. Appropriate cooking/washing of food	Yes	128 (69.2%)	71 (70.2%)	57 (67.8%)	0.873
	f. Avoiding the burden of land near homes and playgrounds with dog feces	Yes	106 (57.3%)	58 (57.4%)	48 (57.1%)	0.656
	g. Avoidance of contact with infected person	No	153 (82.7%)	91 (90.0%)	62 (73.8%)	0.077
	h. Informing the population about these diseases	Yes	133 (71.9%)	74 (73.2%)	59 (70.2%)	0.87

All variables are presented as absolute (Ν) and relative frequencies (%).

**Table 5:** Zoonotic disease knowledge and animal contact-related attitudes of respondents.

Question	Correct Answer	Pet Owners (*n* = 101)	Non Pet Owners (*n* = 84)	*p*-Value
Q1. Do the residents of Mediterranean region have increased risk of developing echinococcosis?	Yes	15 (14.8%)	12 (14.2%)	0.944
Q2. Children’s contact with soil/sand in areas accessible to dogs/cats increases the risk of echinococcosis and/or toxocariasis?	Yes	64 (63.3%)	57 (67.8%)	0.211
Q3. The contact with which animal is more likely to lead to the development of cystic echinococcosis in humans?	Dog	28 (27.7%)	24 (28.5%)	0.682
Q4. The contact with which animal is more likely to lead to the development of toxocariasis in humans?	Both dogs and cats	22 (21.7%)	23 (27.3%)	0.254
Q5. Which age group has the greatest risk of developing echinococcosis?	All ages	55 (54.4%)	34 (40.4%)	0.141
Q6. Which age group has the greatest risk of developing toxocariasis?	Children	24 (23.7%)	14 (16.6%)	0.332

All variables are presented as absolute (Ν) and relative frequencies (%) of right answers.

**Table 6:** Multifactorial analysis of awareness of the zoonoses echinococcosis and toxocariasis.

Variable	Reference Category	R^2^	Beta Coefficient	SE	*p*-Value
Constant		---	4.443	2.048	0.031
Age (40+)	18–39	0.001	0.06	0.56	0.919
Gender (female)	Male	<0.001	−0.28	0.72	0.695
Education (university)	High School	0.095	2.38	0.65	<0.005
Location of Residence (Other *)	City	0.004	−0.41	0.53	0.443
Pet-owner (yes)	No	<0.001	0.11	0.47	0.822

* The word Other in the variable Location of residence refers to Suburban area/Village/Island, SE: Standard Error.

## Supplementary Materials

The following are available online at https://www.mdpi.com/1660-4601/17/15/5292/s1. Table S1: Descriptive characteristics of study participants; Table S2: Zoonotic disease knowledge and animal contact-related attitudes in relation to pet’s place of residence.

## References

[1] Stull J.W., Peregrine A.S., Sargeant J.M., Weese J.S. (2012). Household knowledge, attitudes and practices related to pet contact and associated zoonoses in Ontario, Canada. BMC Public Health.

[2] Carmena D., Cardona G.A. (2013). Canine echinococcosis: Global epidemiology and genotypic diversity. Acta Trop..

[3] Deplazes P., van Knapen F., Schweiger A., Overgaauw P.A.M. (2011). Role of pet dogs and cats in the transmission of helminthic zoonoses in Europe, with a focus on echinococcosis and toxocarosis. Vet. Parasitol..

[4] Smith B. (2012). The “pet effect”: Health related aspects of companion animal ownership. Aust. Fam. Physician.

[5] Ozioko K.U., Okoye C.I., Obiezue R.N., Agbu R.A. (2018). Knowledge, attitudes, and behavioural risk factors regarding zoonotic infections among bushmeat hunters and traders in Nsukka, southeast Nigeria. Epidemiol. Health.

[6] Ma G., Holland C.V., Wang T., Hofmann A., Fan C.K., Maizels R.M., Hotez P.J., Gasser R.B. (2018). Human toxocariasis. Lancet Infect. Dis..

[7] Taylor M.H., O’Connor P., Keane C.T., Mulvihill E., Holland C. (1988). The expanded spectrum of toxocaral disease. Lancet.

[8] Cooper P.J. (2008). Toxocara canis infection: An important and neglected environmental risk factor for asthma?. Clin. Exp. Allergy.

[9] Pivetti-Pezzi P. (2009). Ocular Toxocariasis. Int. J. Med. Sci..

[10] Deshayes S., Bonhomme J., de La Blanchardière A. (2016). Neurotoxocariasis: A systematic literature review. Infection.

[11] Craig P.S., McManus D.P., Lightowlers M.W., Chabalgoity J.A., Garcia H.H., Gavidia C.M., Gilman R.H., Gonzalez A.E., Lorca M., Naquira. C. (2007). Prevention and control of cystic echinococcosis. Lancet Infect. Dis..

[12] Baneth G., Thamsborg S.M., Otranto D., Guillot J., Blaga R., Deplazes P., Solano-Gallego L. (2016). Major Parasitic Zoonoses Associated with Dogs and Cats in Europe. J. Comp. Pathol..

[13] Kern P., Menezes da Silva A., Akhan O., Müllhaupt B., Vizcaychipi K.A., Budke C. (2017). The Echinococcoses. Advances in Parasitology.

[14] World Health Organization (2012). Accelerating Work to Overcome the Global Impact of Neglected Tropical Diseases: A Roadmap for Implementation. http://whqlibdoc.who.int/hq/2012/WHO_HTM_NTD_2012.1_eng.pdf.

[15] Larrieu E., Gavidia C.M., Lightowlers M.W. (2019). Control of cystic echinococcosis: Background and prospects. Zoonoses Public Health.

[16] Bingham G.M., Budke C.M., Slater M.R. (2010). Knowledge and perceptions of dog-associated zoonoses: Brazos County, Texas, USA. Prev. Vet. Med..

[17] Pereira A., Martins Â., Brancal H., Vilhena H., Silva P., Pimenta P., Diz-Lopes D., Neves N., Coimbra M., Alves A.C. (2016). Parasitic zoonoses associated with dogs and cats: A survey of Portuguese pet owners’ awareness and deworming practices. Parasites Vectors.

[18] Ugbomoiko U.S., Ariza L., Heukelbach J. (2008). Parasites of importance for human health in Nigerian dogs: High prevalence and limited knowledge of pet owners. BMC Vet. Res..

[19] Papavasilopoulos V., Bonatsos G., Elefsiniotis I., Birbas C., Panagopoulos P., Trakakis E. (2016). Seroepidemiological investigation of Toxocara canis in a female Greek pregnant population in the area of Athens. Clin. Exp. Obstet. Gynecol..

[20] Theodoridis I., Frydas S., Papazahariadou M., Hatzistilianou M., Adamama-Moraitou K.K., Di Gioacchino M., Felaco M. (2001). Toxocarosis as zoonosis. A review of literature and the prevalence of *Toxocara canis* antibodies in 511 serum samples. Int. J. Immunopathol. Pharmacol..

[21] Papavasilopoulos V., Pitiriga V., Birbas K., Elefsiniotis J., Bonatsos G., Tsakris A. (2018). Soil contamination by Toxocara canis and human seroprevalence in the Attica region, Greece. Germs.

[22] Kostopoulou D., Claerebout E., Arvanitis D., Ligda P., Voutzourakis N., Casaert S., Sotiraki S. (2017). Abundance, zoonotic potential and risk factors of intestinal parasitism amongst dog and cat populations: The scenario of Crete, Greece. Parasit Vectors.

[23] Petropoulos A.S., Chatzoulis G.A. (2019). Echinococcus Granulosus in Childhood: A Retrospective Study of 187 Cases and Newer Data. Clin. Pediatr..

[24] Seimenis A. (2003). Overview of the epidemiological situation on echinococcosis in the Mediterranean region. Acta Trop..

[25] Sotiraki S., Chaligiannis I. (2013). Cystic echinococcosis in Greece. Past and present. Parasite.

[26] Chen J., Liu Q., Liu G.H., Zheng W., Bin Hong S.J., Sugiyama H., Zhu X.-Q., Elsheikha H.M. (2018). Toxocariasis: A silent threat with a progressive public health impact. Infect. Dis. Poverty.

[27] Awosanya E.J., Akande H.O. (2015). Animal health care seeking behavior of pets or livestock owners and knowledge and awareness on zoonoses in a university community. Vet. World.

[28] Woodhall D.M., Garcia A.P., Shapiro C.A., Wray S.L., Shane A.L., Mani C.S., Stimpert K.K., Fox L.M., Montgomery S.P. (2017). Assessment of U.S. pediatrician knowledge of toxocariasis. Am. J. Trop. Med. Hyg..

[29] Palmer C.S., Robertson I.D., Traub R.J., Rees R., Andrew Thompson R.C. (2010). Intestinal parasites of dogs and cats in Australia: The veterinarian’s perspective and pet owner awareness. Vet. J..

[30] Dakkak A. (2010). Echinococcosis/hydatidosis: A severe threat in Mediterranean countries. Vet. Parasitol..

[31] Woodhall D.M., Eberhard M.L., Parise M.E. (2014). Neglected parasitic infections in the United States: Toxocariasis. Am. J. Trop. Med. Hyg..

[32] https://www.esccap.org/uploads/docs/2fe4poh6_0778_ESCCAP_Guideline_GL1_v10_1p.pdf..

[33] Nijsse R., Ploeger H.W., Wagenaar J.A., Mughini-Gras L. (2016). Prevalence and risk factors for patent Toxocara infections in cats and cat owners’ attitude towards deworming. Parasitol. Res..

[34] Matos M., Alho A.M., Owen S.P., Nunes T., Madeira de Carvalho L. (2015). Parasite control practices and public perception of parasitic diseases: A survey of dog and cat owners. Prev. Vet. Med..

[35] McNamara J., Drake J., Wright I. (2018). Survey of German pet owners quantifying endoparasitic infection risk and implications for deworming recommendations. Parasites Vectors.

[36] Bourguinat C., Keller K., Bhan A., Peregrine A., Geary T., Prichard R. (2011). Macrocyclic lactone resistance in Dirofilaria immitis. Vet. Parasitol..

[37] Geary T.G., Bourguinat C., Prichard R.K. (2011). Evidence for macrocyclic lactone anthelmintic resistance in Dirofilaria immitis. Top. Companion Anim. Med..

[38] Kopp S.R., Kotze A.C., McCarthy J.S., Coleman G.T. (2007). High-level pyrantel resistance in the hookworm Ancylostoma caninum. Vet. Parasitol..

[39] Castro P.D.J., Howell S.B., Schaefer J.J., Avramenko R.W., Gilleard J.S., Kaplan R.M. (2019). Multiple drug resistance in the canine hookworm Ancylostoma caninum: An emerging threat?. Parasites Vectors.

[40] Chelladurai J.J., Kifleyohannes T., Scott J., Brewer M.T. (2018). Praziquantel resistance in the zoonotic cestode Dipylidium caninum. Am. J. Trop. Med. Hyg..

[41] Raza A., Rand J., Qamar A.G., Jabbar A., Kopp S. (2018). Gastrointestinal parasites in shelter dogs: Occurrence, pathology, treatment and risk to shelter workers. Animals.

